# Implementing the framed portrait experience with Italian breast cancer survivors: a pilot study assessing short term effects of an existential approach to body image, coping skills, and self-efficacy

**DOI:** 10.1007/s11764-023-01438-6

**Published:** 2023-08-19

**Authors:** Denise Vagnini, Antonia Sorge, Chiara Acquati, Francesca Antonia Colafemmina, Emanuela Saita

**Affiliations:** 1 Department of Psychology, Università Cattolica del Sacro Cuore, Milan, Italy; 2 Graduate College of Social Work, University of Houston, Houston, TX USA; 3 Department of Clinical Sciences, Tilman J. Fertitta Family College of Medicine, University of Houston, Houston, TX USA; 4 Department of Health Disparities Research, The UT MD Anderson Cancer Center, Houston, TX USA

**Keywords:** Body image, Breast cancer survivors, Women, Coping, Psychosocial intervention, Self-efficacy

## Abstract

**Purpose:**

Breast cancer (BC) and its treatments significantly impact the psychological wellbeing of women. Interventions offered during cancer survivorship have documented positive consequences for quality of life. However, limited evidence is available regarding the implementation of therapeutic photography. This study investigated the efficacy of the framed portrait experience (FPE) when implemented to BC survivors.

**Methods:**

A quasi-experimental study was conducted. Participants were enrolled in a non-randomized pre-post intervention with a comparison group. Forty BC survivors were recruited using a convenience sampling approach; of these, 20 were subsequently allocated to the intervention (FPE group) and 20 to the comparison group. Participants were assessed at pretest and posttest (3 weeks later) using self-reported measures of body image, coping, self-esteem, and self-efficacy. Independent samples *t*-tests compared group composition at pretest. Mixed between-within 2 × 2 repeated-measures ANOVAs examined pretest–posttest changes in the variables of interest.

**Results:**

No differences were detected between groups at pretest. A significant interaction effect on body image, problem-focused coping, emotion-focused coping, and in self-efficacy competence subscale (*p* < 0.05) was identified. Post hoc pairwise comparisons with the Bonferroni correction indicated improvement on these domains in the FPE group vs. comparison group. Additionally, significant main effects of time on self-efficacy total score and magnitude subscale (*p* < 0.05) were found.

**Conclusions:**

Preliminary results support the efficacy of FPE, but further research is needed.

**Implications for Cancer Survivors:**

Existential approaches inclusive of self-portraits and illness narratives can be utilized to support BC survivors in the management of the psychological consequences of the illness.

## Background

Breast cancer (BC) is the most common form of cancer diagnosed in women worldwide. In Italy, BC represents the most common neoplasm identified in females. In 2022, about 55.700 new diagnoses were reported, with a 0.5% increase compared to 2020 [1]. Despite significant improvements in treatments and overall survival, the long-term adverse impact of the illness contributes to impaired quality of life, physical functioning, impaired femininity, and sexual health [2]. Among the domains most affected by the illness, the body image is susceptible to many changes (e.g., scars, alteration in the shape of the breast, and hair loss). A growing body of literature underscores how the experience of BC modifies the general sense of self and perception of body image over time [3], especially in women who internalized traditional gender roles, who engaged in self-surveillance, and experienced body shame [4, 5].

Body image is defined as perceptions, thoughts, or emotions about one’s physical appearance [6]. Both patients (17–33%) and long-term survivors (15–30%) report body image issues [7, 8]. Specifically, a poor body image perception negatively influences sexuality and social functioning, in addition to quality of life [9]. Moreover, literature has documented how body image discomfort compromises self-efficacy and self- esteem [10]. Despite growing interest in this domain of quality-of-life, empirical support for interventions dedicated to breast cancer survivors is still lacking [11, 12]. To contribute to the limited body of evidence that has investigated intervention approaches to enhance body image in this group, this pilot study evaluated the preliminary efficacy of the framed portrait experience (FPE) [13–15] among midlife breast cancer survivors.

### The framed portrait experience (FPE)

The FPE is rooted in three guiding principles. First, cancer and its treatments are considered an affect-laden experience [13]. Second, the intervention utilizes non-verbal stimuli to enhance the verbal production [16]. Specifically, a photographic portrait allows the individuals to be in contact with their own self and facilitates the construction of an illness narrative. Several studies have shown support for the use of photography in clinical settings [17]. For example, Frith et al. [18] have reported that self-portraits strengthened the ability to cope with cancer and enhanced positive self-representations. Photos linked to personal experiences activate attention more than neutral photo [19], so the portraits can become prompts for autobiographical narration, in order to understand how women have internalized and elaborated the illness and to characterize their efforts at recovery and adaptation [20]. The FPE integrates characteristics derived from both reenactment therapy and therapeutic photography [21]. In fact, similarly to what occurs in re-enactment phototherapy, the images (i.e., the portraits) are co-constructed with the patient. In the FPE, pictures are helpful to construct a narrative about the illness’ impact in the person’s life story, and this is one of the strengths of the therapeutic photography.

### The current intervention

**Fig. 1:**
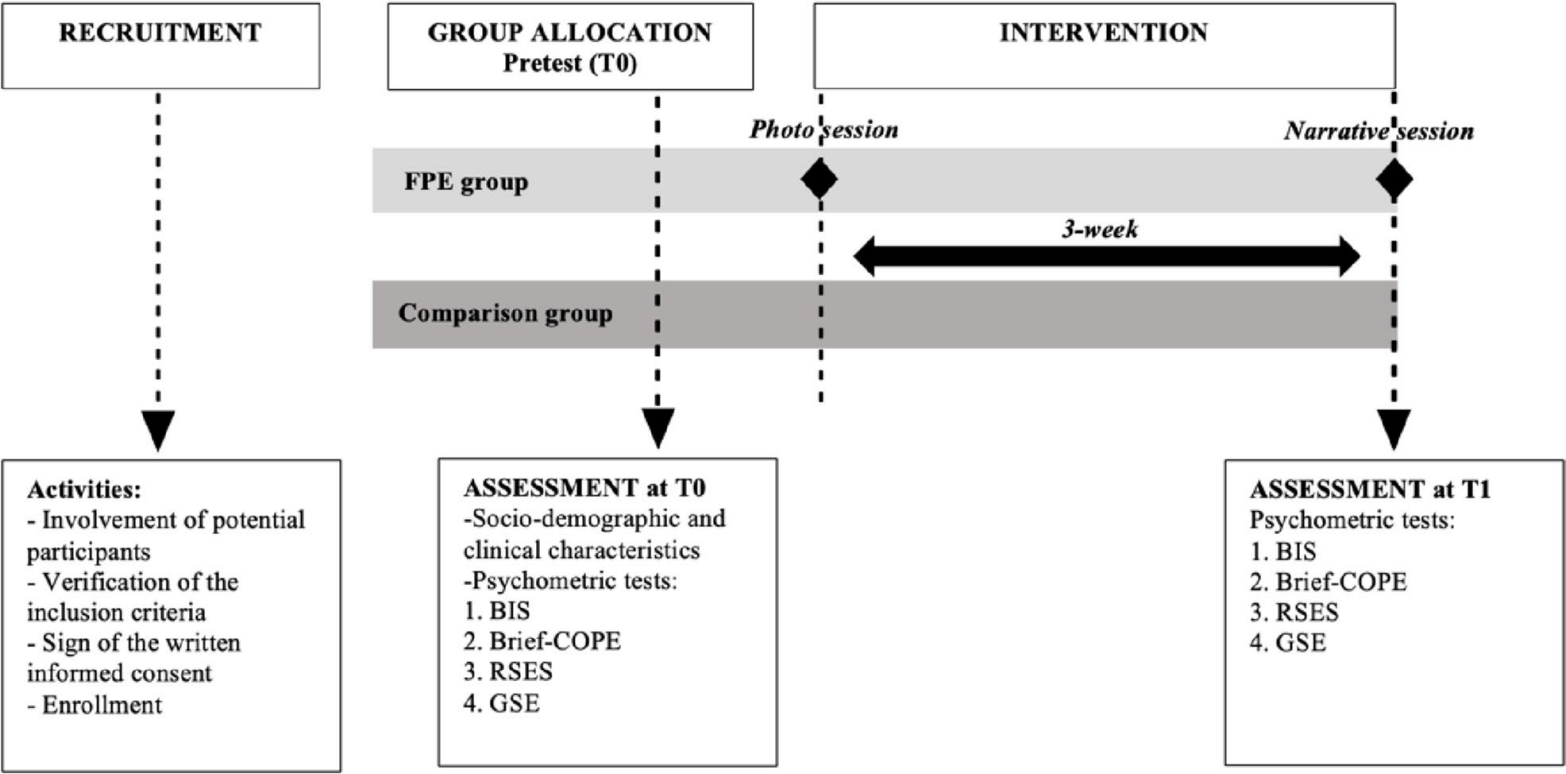
Synthesis of the framed portrait intervention. Abbreviations: FPE, framed portrait experience; BIS, body image scale; Brief-COPE, Coping Orientation toward Problems Experienced questionnaire; RSES, Rosenberg Self-esteem Scale; GSE, General Self-Efficacy Scale

The intervention was organized in two separate sessions: a photographic session and a narrative session with a psychologist. Pretest assessment took place before starting the photographic session, while the posttest data collection occurred at the end of the narrative session (or after 3 weeks for the comparison group who was not involved in the intervention). In session 1, a specialized professional took photographs of the patient that evoked a symbolic representation of the past, which is organized around the diagnosis with cancer; the present (after the end of treatments); and the future self that the patient imagines and/or wants to reach. Each moment is captured in 3 or 4 images. In preparation for the second session, 1–2 photographs from each collection are printed and presented to the participant to elicit their own life story utilizing memories, emotions, and feelings produced by the portraits [13]. Participants are then asked to give a title to the photographs. This intervention aims to assist women to elaborate the cancer experience, to “reorganize” the event in their life story, and to reflect on the changes caused by the disease. For a more detailed presentation of the framed portrait experience please review Saita et al. [13–15]. Figure 1 shows the timeline of the intervention.

### Examples from the Framed Portrait Experience (FPE)

**Fig. 2.**
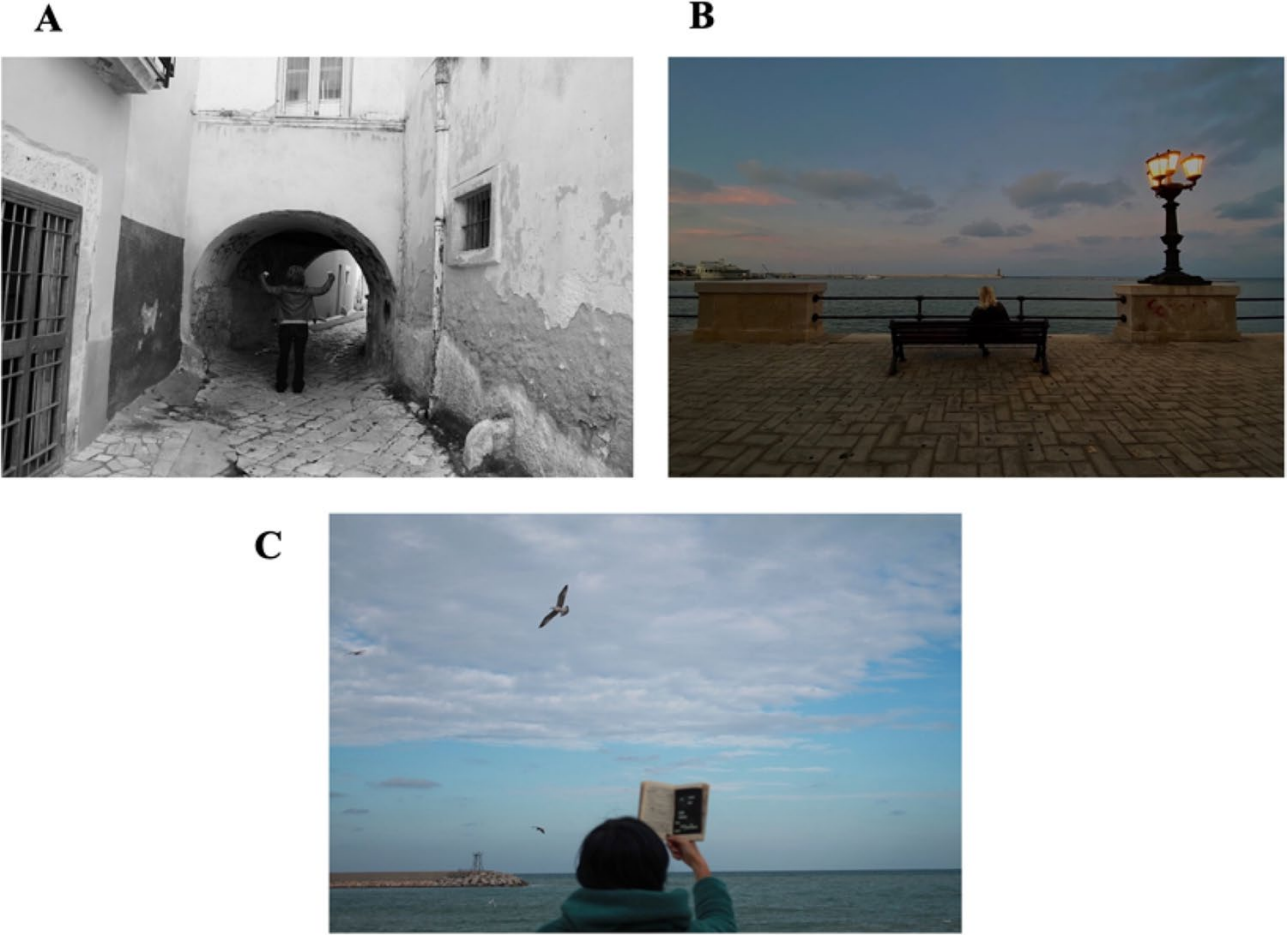
Representations of: “*The past*” (portrait “A”), *“The present*” (portrait “B”), and “*The future*” (portrait “C”). *Portrait “A”*: for the first set of photographs relative to the past, *Adele* stated that receiving the diagnosis of breast cancer was like entering in a long black tunnel. The world fell on her shoulders. The loss of hair and eyelashes was compared to the loss of identity. Despite these experiences, *Adele* is photographed with her head held high and shoulders straight, while staring at the path in front of her. *Adele* stated that she has always sought strength to overcome problems and to support her loved ones. *Portrait “B”*: to describe her present life, *Mary* chose to be photographed while watching *“a sunset, at peace*”. For *Mary* the sunset represents an acknowledgement to the day that has just been lived, hoping to see a new dawn. Sunrise and sunset are her favorite moments of the day, since she has learned to appreciate the *"here and now"* and to give value to the words *“see you tomorrow”.* Finally, *Portrait “C”*: *Clara* resumed writing poetry while undergoing her cancer treatment, a time when her inspiration came in a sudden, unexpected, and spontaneous way. In this photograph, which symbolizes her expectations for the future, she holds in her hand a book that she would like to publish

Figure 2 reports some examples of portraits from photographic sessions with breast cancer survivors. For each photograph, a summary of the key concepts addressed is reported. Fictional names, photos with not recognizable faces and details have been utilized. Additionally, the authors have anonymized personal details of the stories and did not include participants’ sequence of portraits to protect their privacy.

### Purpose and Hypothesis

The current study investigates the preliminary efficacy of the FPE as an intervention aimed at enhancing the psychological health of BC survivors. Specifically, it was hypothesized (H1) that lower body image concerns and active coping strategies would be reported by BC survivors in the intervention group. Additionally, it was hypothesized (H2) that BC survivors who completed the intervention would report higher scores on self-esteem and self-efficacy, similarly to findings from a previous study conducted with adolescent and young adult cancer survivors [13].

## Methods

### Study design

A quasi-experimental study was conducted to assess the preliminary efficacy of FPE on psychological health outcomes of BC survivors.

Participants were enrolled in a non-randomized pre-post intervention with a comparison group. Specifically, two groups were involved: an intervention group, composed of women who participated in the photographic and narrative sessions of the FPE, and a comparison group in a waitlist, so that they had the opportunity to participate in the FPE at the end of the intervention (i.e., 3 weeks later). The efficacy of the intervention was measured by a pre-posttest comparison of the selected measures.

### Procedure and participants

Participation in the study was voluntary but limited to the following inclusion criteria: women, aged ≥ 18 years, native Italian speakers, diagnosed with BC, and who had completed their cancer treatments.

Sample size was calculated using G*Power Software 3.1 [22]. Setting *α* = 0.05 and power (1-β err prob) of 0.80, a total sample of *N* = 40 was required to detect a small effect size according to criteria for Cohen’s *d* [23].

Participants were invited to the study using a convenience sampling approach. Patients who met the inclusion criteria were provided with detailed information about the study and the intervention offered as part of the research project. Forty-five women were contacted. Investigators partnered with a non-profit association advocating for psycho-physical rehabilitation for BC survivors who helped to contact the first *n* = 23 women to whom the study was presented, while the remaining *n* = 22 were reached through snowball sampling. After a screening for eligibility, forty women accepted to be enrolled in the study and written informed consent was obtained by each of them. Once the a priori sample size was reached, recruitment stopped. As a sign of gratitude and appreciation for their time, participants from the FPE group received a printed copy of the portraits.

The study was performed in accordance with the ethical standards as laid down in the 1964 Declaration of Helsinki and its later amendments or comparable ethical standards. Approval was obtained from the Ethics Commission for Research in Psychology (*CERPS: Commissione Etica per la Ricerca in Psicologia*) of the Department of Psychology at Università Cattolica del Sacro Cuore of Milan (Italy), protocol no. 46-23.

### Instruments

A printed data sheet was used to collect detailed socio-demographic and clinical characteristics at pretest. Self-reported questionnaires were administered before and after the intervention. The Italian version of the *Body Image Scale (BIS)* [24, 25] examined affective, cognitive, and behavioral self-perceptions of body image changes related to the disease and treatment. It consists of 10 items rated on a 4-point Likert scale (from 0 = not at all; to 3 = very much). A total score between 0 (non-compromised body image) and 30 (highly impaired body image) is obtained. BIS is specifically validated with a sample of patients with BC (internal consistency coefficient: *α* = 0.916); however, since the scientific literature does not provide a cut-off for clinical interpretation, we created 3 *ad hoc* classes labelled "Good body image" (scores 0–10), “Composite body image” (scores 11–20), and “impaired body image” (scores 21–30), as previously implemented by the authors in earlier works [26].

The *Brief-COPE, Coping Orientation toward Problems Experienced questionnaire* [27] is designed to measure how individuals cope with stressful life events, and it is often used in health-care settings. It consists of 28 items divided in 14 2-item subscales with good internal consistency, rated on a 4-point Likert-style scale (from 1 = I haven't been doing this at all; to 4 = I’ve been doing this a lot). These subscales can be further grouped into broad categories. Three overarching dimensions considered in the present study, and previously investigated [28], are described below. “Problem focused coping” summarizes facets referring to active coping, use of information support, planning, and positive reframing; high score indicates a hands-on approach to problem solving. “Emotion focused coping” is characterized by venting, use of emotional support, humor, acceptance, self-blame, and religion; a high score shows ability to regulate emotions associated with the stressor. “Avoidant coping” is characterized by self-distraction, denial, substance use, and behavioral disengagement; a high score describes physical or cognitive efforts to disengage from the stressor situation, instead low scores are indicative of respondent’s adaptive coping skills.

The Italian version of the *Rosenberg Self-Esteem Scale (RSES)* [29, 30] was used to assess patients’ self-esteem. The instrument consists of 10 items, and the respondents are asked to indicate their level of agreement on a 4-point Likert-type scale (from 1 = strongly disagree; to 4 = strongly agree). It has a high internal consistency, with *α* = 0.84.

The Italian version of *General Self-Efficacy Scale (GSE)* [31, 32] was utilized to measure one’s belief to reach a goal (i.e., perception of self-efficacy). This instrument consists of 17 items scored on a 5-point Likert scale (from 1 = strongly disagree; to 5 = strongly agree), and it showed good internal consistency with Cronbach’s alpha comprised between 0.64 and 0.74. The scale can be used as mono-dimensional or can allow the detection of three main components (subscales) which refer to Bandura’s socio-cognitive theory [33]: “Magnitude”, measuring the perceived efficacy as regards levels of performance difficulty; “Strength,” as the ability to persevere and cope with obstacles; and finally, “Competence,” referring to a sense of global proficiency in facing problems.

### Statistical analysis

Data were analyzed using IBM SPSS Statistics 27.0. Descriptive statistics (i.e., frequency, mean, and SD) and comparison tests (i.e., *χ*^2^ and independent *t*-test, *α* = 0.05 two-tailed) were performed to present socio-demographic variables and clinical characteristics of the sample. Independent sample *t*-tests (*p* < 0.05) were conducted to assess between-groups differences at pretest. Cohen’s *d* effect sizes were provided according to criteria (*d* = 0.2 small effect; *d* = 0.5 medium effect; *d* = 0.8 large effect) [23].

Then, a series of mixed between-within 2 (groups: FPE group vs. comparison group) × 2 (assessment times: pretest vs. posttest) repeated measures analyses of variance (ANOVAs) were conducted to compare the two groups at the two measurement timepoints: pretest (T0), posttest (T1: 3 weeks) on body image (BIS), problem-focused coping (Brief-COPE subscale), emotion focused coping (Brief-COPE subscale), avoidant coping (Brief-COPE subscale), self-esteem (RSES), self-efficacy (GSE), magnitude (GSE subscale), strength (GSE subscale), and competence (GSE subscale). Effect size indicators (partial Eta-squared: *η*^2^_partial_) were used to quantify the global difference of the two groups across time. Scores were interpreted with the following benchmarks: small (η^2^_partial_ = 0.01), moderate (η^2^_partial_ = 0.06), and large (η^2^_partial_ = 0.14) [23]. Where interaction effects of the mixed ANOVAs were found to be significant, post-hoc pairwise comparisons with Bonferroni correction were performed.

## Results

### Descriptive statistics

Table 1 provides socio-demographic and clinical characteristics by group. No significant (*p* < 0.05) differences were highlighted, except for the type of surgery: women in FPE group were equally divided between those who had breast-conserving surgery and mastectomy; instead, more participants of comparison group had mastectomy. All participants (*N* = 40) completed all survey questionnaires without missing responses.

**Table 1:** Sociodemographic and clinical characteristics of intervention (*n* = 20) and comparison (*n* = 20) groups

	Intervention group (*n* = 20)	Comparison group (*n* = 20)	
Age, *M *± SD (range)	56.55 ± 11.48 (38-76)	52.40 ± 9.93 (34-74)	t_(38)_ = 1.223, *p =* 0.322
Marital status, no. (%)			
Unmarried	3 (15)	4 (20)	
Married	9 (45)	13 (65)	*χ*^2^_(3)_ = 3.156, *p* = 0.368
Divorced	3 (15)	1 (5)	
Widow	5 (25)	2 (10)	
Educational level, no. (%)			
Elementary school	1 (5)	0	
Middle school	3 (15)	3 (15)	*χ*^2^_(3)_ = 1.381, *p* = 0.710
High school diploma	11 (55)	10 (50)	
University degree	5 (25)	7 (35)	
Working status, no. (%)			
Employed	13 (65)	13 (65)	*χ*^2^_(2)_ = 4.667, *p* = 0.097
Unemployed	2 (10)	6 (30)	
Retired	5 (25)	1 (5)	
Surgery type, no. (%)			
Breast-conserving surgery	10 (50)	3 (15)	χ^2^_(1)_ = 5.584, *p* = 0.018*
Mastectomy	10 (50)	17 (85)	
Surgery side, no. (%)			
Monolateral	19 (95)	19 (95)	
Bilateral	1 (5)	1 (5)	*χ*^2^_(1)_ = 0.000, *p =* 0.999
Surgery year, no. (%)			
2012–2016	12 (60)	6 (30)	*χ*^2^_(1)_ = 2.702, *p* = 0.100
2017–2022	8 (40)	14 (70)	
Treatments, no. (%)			
Chemotherapy	2 (10)	1 (5)	
Hormonal therapy	5 (25)	7 (35)	
Chemo + hormonal therapy	2 (10)	2 (10)	
Chemo + radiotherapy	2 (10)	3 (15)	*χ*^2^_(6)_ = 3.200, *p* = 0.783
Radio + hormonal therapy	2 (10)	1 (5)	
Chemo + radio + hormonal therapy	6 (30)	3 (15)	
No treatment	1 (5)	3 (15)	
Body image scale (BIS)—*Ad hoc* classes, no. (%)	T0; T1	T0; T1	
Good body image (scores 0–10)	7 (35); 10 (50)	5 (25); 4 (20)	
Composite body image (scores 11–20)	5 (25); 7 (35)	4 (20); 5 (25)	*χ*^2^_(2)_ = 0.918, *p* = 0.632
Impaired body image (scores 21–30)	8 (40); 3 (15)	11 (55); 11 (55)	

^*^p < 0.05

### Pretest comparison between intervention group (*n* = 20) and comparison group (*n* = 20) on psychological measures

No significant (*p* < 0.05) baseline differences were found between the groups on body image (*t*_(38)_ = – 0.694, *p* = 0.492, Cohen’s *d* = – 0.219); coping problem-focused (*t*_(38)_ = 0.538, *p* = 0.594, Cohen’s *d* = 0.170); coping emotion-focused (*t*_(38)_ = – 0.290, *p* = 0.774, Cohen’s *d* = – 0.092); avoidant coping (*t*_(38)_ = – 0.600, *p* = 0.552, Cohen’s *d* = – 0.190); self-esteem (*t*_(38)_ = 0.052, *p* = 0.959, Cohen’s *d* = 0.016); self-efficacy (*t*_(38)_ = 0.542, *p* = 0.591, Cohen’s *d* = 0.171); self-efficacy magnitude subscale (*t*_(38)_ = 0.147, *p* = 0.884, Cohen’s *d* = 0.047); self-efficacy strength subscale (t_(38)_ = 0.890, *p* = 0.379, Cohen’s d = 0.281); and self-efficacy competence subscale (t_(38)_ = 0.158, *p* = 0.875, Cohen’s d = 0.050).

### Mixed 2 × 2 ANOVA’s models

The interaction effects Time*Group were significant with *p *< 0.05 on body image (F_(1,38)_ = 18.784, *p < *0.001, η^2^_partial_ = 0.331, observed power = 0.988), problem-focused coping (F_(1,38)_ = 9.729, *p* = 0.003, η^2^_partial_ = 0.204, observed power = 0.860), emotion focused coping (F_(1,38)_ = 7.375, *p* = 0.010, η^2^_partial_ = 0.163, observed power = 0.754), and self-efficacy competence subscale (F_(1,38)_ = 7.549, *p* = 0.009, η^2^_partial_ = 0.166, observed power = 0.764). These findings indicated that the difference between T0 (pretest) and T1 (posttest: 3-week) was different between FPE group and comparison group.

**Fig. 3:**
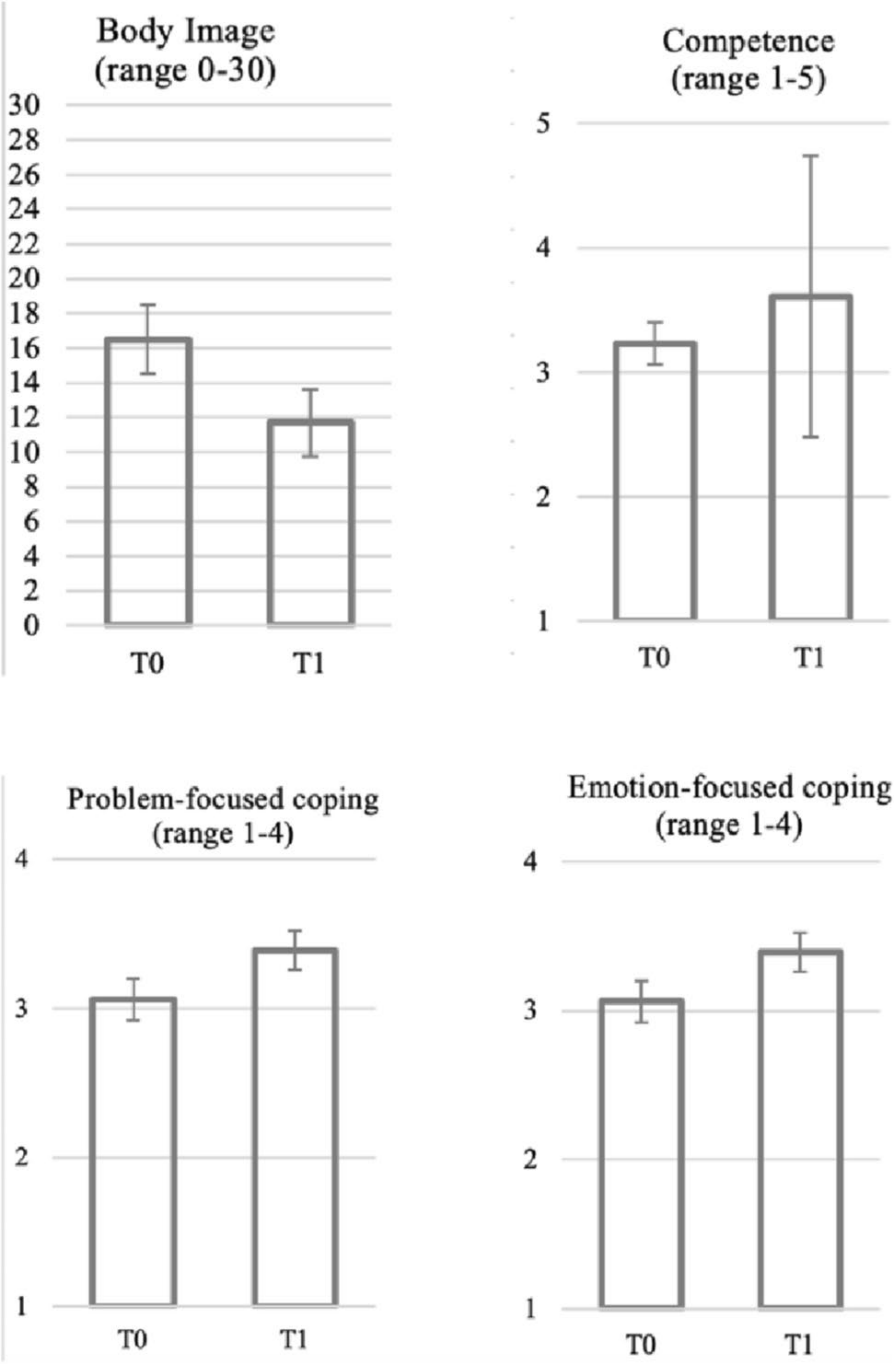
Pretest and posttest FPE group mean scores (variability = SD) on significant psychological variables

Post-hoc pairwise comparisons with Bonferroni correction showed a significant difference between the assessments at T0 and T1 in the FPE group. Specifically, body image concerns significantly decreased from T0 (*M* = 16.50, SD = 1.99) to T1 (*M* = 11.70, SD = 1.91), problem-focused coping skills improved from T0 (*M* = 3.06, SD = 0.14) to T1 (*M* = 3.39, SD = 0.13), emotion-focused coping skills improved from T0 (*M* = 3.03, SD = 0.09) to T1 (*M* = 3.34, SD = 0.10), and self-efficacy competence subscale showed an improvement from T0 (*M* = 3.23, SD = 0.17) to T1 (*M* = 3.61, SD = 1.13). The comparison group did not show significant changes between the two data collection points on body image concerns (*p* = 0.387), problem-focused coping (*p* = 0.566), emotion-focused coping (*p* = 0.714), and self-efficacy competence subscale (*p* = 0.660) (Fig. 3).

Table 2 presents the interaction effects (Time*Group) from the 2 × 2 mixed between-within subjects ANOVA’s models comparing FPE group and comparison group on the variables of interest.

**Table 2:** Interaction effects of time*group for mixed between-within subjects ANOVAs

	Interaction effects			
	*F*_(df)_	*p value*	*η*^2^_partial_^†^	Observed power
Body image (BIS)	18.784_(1,38)_	0.0001**	0.331	0.988
Problem-focused subscale (Brief-Cope)	9.729_(1,38)_	0.003*	0.204	0.860
Emotion-focused subscale (Brief-Cope)	7.375_(1,38)_	0.010*	0.163	0.754
Avoidant subscale (Brief-Cope)	1.085_(1,38)_	0.304	0.028	0.174
Rosenberg Self-esteem Scale (RSES)	0.891_(1,38)_	0.351	0.023	0.151
Self-Efficacy Scale—total score (GSE)	0.276_(1,38)_	0.603	0.007	0.081
Self-Efficacy—Magnitude subscale (GSE)	2.216_(1,38)_	0.145	0.055	0.306
Self-Efficacy—Strength subscale (GSE)	2.912_(1,38)_	0.096	0.071	0.384
Self-Efficacy—Competence subscale (GSE)	7.549_(1,38)_	0.009*	0.166	0.764

^**† **^*η*^2^_partial_, Effect size: small (*η*^2^_partial_ = 0.01); moderate (*η*^2^_partial_ = 0.06); and large (*η*^2^_partial_ = 0.14) [23]

* *p *< 0.05, ** *p *< 0.001

Finally, contrary to the stated hypothesis, analyses showed a significant main effect of time (i.e., the within variable) on total score for self-efficacy (*F*_(1,38)_ = 4.809, *p* = 0.034, *η*^2^_partial_ = 0.112, observed power = 0.570), and on the self-efficacy magnitude subscale (*F*_(1,38)_ = 8.864, *p* = 0.005, *η*^2^_partial_ = 0.189, observed power = 0.827). Specifically, self-efficacy total score and the self-efficacy magnitude subscale improved between the two data collection points. This means that the present sample reported a positive change on these dimensions, but that this improvement was independent of the group allocation.

## Discussion

BC triggers significant psychological impairments in patients and women are faced with existential life-and-death issues. The adaptation to the illness requires the individuals to integrate the cancer experience in their life narrative. However, this process can be challenging as many patients experience difficulties in the verbal expression of emotions and tend to suppress feelings [34]. Innovative psychosocial interventions aimed at improving the psychological health of patients must consider this aspect and propose new means through which participants can elaborate the illness experience and achieve better quality of life during survivorship. Visual methods (e.g., the use of photographs) are increasingly proposed in psychosocial interventions to make meaning of the event [18, 35]. Images appear to an effective medium to assist patients conveying messages that they would not be able to express in their own words. Photographic techniques, combined with narrative ones, can facilitate the individual’s ability to recognize and express emotions and foster the processing of emotionally meaningful experiences, such as the oncological disease [14].

Firstly, results showed that the FPE has contributed to increased self-satisfaction. According to Erik Erikson [36], the presence of a positive self-identity is manifested when the individual “feels at home inhabiting the body” and experiences a high degree of inner self-satisfaction. Likewise, the “Theory of the discrepancy of the Self” by Tory Higgins [37] states that a discrepancy between the actual perception of the self and the ideal self (i.e., how the person wish to be) generates contrasting emotions, which can be contained if this gap is reduced; in this case through specifically targeted interventions aimed at the improvement of this dimension. Hence, it appears that the FPE has contributed to increased self-satisfaction in the present sample. Findings showed lower scores on body image scale, meaning that patients experienced a better global perception of themselves at the end of the study.

Second, emotion- and problem-focused coping skills significantly improved for the intervention group. These findings are consistent with previous results documented in the literature. A patient-centered approach promotes better adaptation of the individual, which actively contributes to one’s decision-making [38]. Moreover, the meaning-making process derived from the re-organization of events in the life story has been associated with better coping skills and adjustment [20]. These two outcomes were both consistent with our first hypothesis, according to which we expected to observe an improvement on body image perception and coping strategies for women in FPE group unlike the comparison group.

Then, in contrast with our second hypothesis we did not observe an improvement in self-esteem. This could be explained by the different targets considered by the two studies (adolescents and young cancer survivors vs. adult middle-aged breast cancer survivors). Since AYAs are confronted with psychosocial transitions while facing cancer [39], their self-esteem is continuously questioned and negotiated in different contexts and systems (i.e., medical settings, educational, and working environments, peers and partnered relationships, family functioning), compared to cancer survivors who are adults already. This consideration can be of assistance in explaining why an intervention like the FPE tends to have a greater impact on self-esteem of adolescents and young adults, who are going through a new phase of the life cycle with the assumption of new social and family roles and the discovery and affirmation of themselves. However, further studies are need to confirm this observation.

Finally, results showed an improvement in self-efficacy in terms of “perceived competence” (i.e., the perception of having the competence to face a difficulty). This result is partially consistent with our second hypothesis and the previous study from the same authors [13] where both the self-efficacy total score and the competence subscale improved after the intervention. However, this preliminary finding is critical since the scientific literature considers self-efficacy as a crucial psychological resource and given previous evidence supporting that gains in self-efficacy promote improvements in other health-related dimensions [40]. Summarizing the present findings (i.e., enhancement on body image perception, problem-focused and emotion-focused coping abilities, and perceived competence as a facet of self-efficacy), it seems that when BC survivors are invited to confront a private experience through the narratives elicited by portraits, they are better able to make meaning of the cancer experience [35]. This process of self-understanding, re-elaboration of own’s story and meaning making underpins the functional elaboration of the BC experience, and it seems to be closely linked to the improvement of overall psychological health.

### Study limitations

Several limitations are present. First, the sampling strategy and the small sample size affect the generalizability of the present contribution. Further, we had to consider that BC survivors who deliberately choose to take part in such of interventions, may have distinctive inner characteristics from those who do not offer themselves as participants in the research. This has also been observed in previous studies who hypothesized on the one hand a possible intrinsic motivation and autonomy [41], on the other hand maybe a generalized need for support, not explicit in a specific request and in a specific context [12]. For these reasons, future research should consider these aspects to understand how to overcome this bias. Second, the lack of randomization did not allow the authors to control for selection bias. Third, the absence of a follow-up evaluation prevented the research team from investigating the effects of the intervention over time. In addition, it is necessary to be cognizant that an increase in self-efficacy was independent of the intervention condition.

Future studies with a larger sample and using experimental designs are necessary to address these aspects. Finally, women in the comparison group were more likely to have undergone mastectomy than women in the FPE group. Differences in the type of surgery may affect the psychological well-being of patients with BC [42] and have contributed to present results.

## Clinical implications and conclusions

Efforts to support the psychological recovery of BC cancer survivors can be strengthened by the application of an effective, easily implemented, and low-cost treatment approach [13]. While this represents a preliminary investigation, it is important to emphasize that the FPE possesses all these characteristics. Results of this study provide emerging evidence supporting that the FPE can improve psychological health outcomes of BC survivors and assists with the integration of the cancer experience within a woman’s own life story.
